# Increasing trends in fecundity and calf survival of bottlenose dolphins in a marine protected area

**DOI:** 10.1038/s41598-018-38278-9

**Published:** 2019-02-11

**Authors:** Barbara J. Cheney, Paul M. Thompson, Line S. Cordes

**Affiliations:** 1 University of Aberdeen, Institute of Biological and Environmental Science, Lighthouse Field Station, Cromarty, IV11 8YL UK; 2 Bangor University, School of Ocean Sciences, Menai Bridge, LL59 5AB UK

## Abstract

Estimates of temporal variation in demographic rates are critical for identifying drivers of population change and supporting conservation. However, for inconspicuous wide-ranging species, births may be missed and fecundity rates underestimated. We address this issue using photo-identification data and a novel robust design multistate model to investigate changes in bottlenose dolphin fecundity and calf survival. The model allows for uncertainty in breeding status, and seasonal effects. The best model estimated an increase in the proportion of females with newborn calves from 0.16 (95% CI = 0.11–0.24) in 2001 to 0.28 (95% CI = 0.22–0.36) in 2016. First year calf survival also increased over this period from 0.78 (95% CI = 0.53–0.92) to 0.93 (95% CI = 0.82–0.98). Second year calf survival remained lower, but also showed an increase from 0.32 (95% CI = 0.19–0.48) to 0.55 (95% CI = 0.44–0.65). Females with newborn calves had a slightly higher mortality than those with older calves, but further work is required to evaluate potential costs of reproduction. This study presents a rare example of empirical evidence of a positive trend in reproduction and survival for a cetacean population using a Marine Protected Area.

## Introduction

Environmental and anthropogenic changes can have direct impacts on the demography and hence dynamics of animal populations^1,2^. As such, accurately estimating temporal variation in demographic rates and assessing their contribution to population abundance are fundamental objectives of many ecological studies and vital for conservation and management^3,4^. The established principle for long-lived species is that population dynamics are most sensitive to variations in adult female survival^5,6^. Reproductive costs in females may increase mortality, reduce offspring survival, and result in trade-offs between current and future reproduction^7–9^. However, those costs may be too small to be detectable, or there may even be a positive relationship between higher female fecundity and survival as a result of individual heterogeneity^8^. As a result, monitoring adult survival may provide limited insights into population responses to environmental variation^6^, whereas reproduction and juvenile survival may show clearer variation in relation to environmental change^6^ and limiting resources^5^. Consequently, understanding trends in reproduction and juvenile survival may provide a more sensitive method to detect impacts of anthropogenic activities or environmental change before these significantly affect population status.

Understanding the demographic drivers of population change can be particularly challenging for wide-ranging inconspicuous species. For cetacean populations, recent studies of demographic rates have been underpinned by long-term individual-based photo-identification studies (e.g. refs^10–12^). Fecundity, here as in other studies defined as reproductive rate, has been estimated in a number of different ways from using the proportion of identifiable reproductive females with newborn^13,14^ or older calves^15,16^, the inverse of the inter-birth interval^12^, modelling inter-birth intervals based on the probability of birth conditional on previous reproductive histories^11,17,18^ or simply reporting the mean inter-birth interval^19–21^. Calf survival to different ages has also been explored by estimating apparent survival rates with capture-recapture models^10,22^.

However, there are two important issues with these approaches. Firstly, they do not account for imperfect detections, which are particularly important for inconspicuous and wide-ranging species, such as cetaceans. Not all individuals will be observed on all occasions (i.e. they may be missed, or not in the survey area) and breeders can be misclassified as non-breeders if young are not detected (i.e. they die before the female is next observed or may not be sighted with the female). In a number of different species early mortality can be highest during the first month following birth (e.g. refs^19,23–25^). Even in an area with exceptionally high sighting probabilities, where all or nearly all adults are seen on every survey, some births were missed^19^. This can result in biased demographic rates^26^, underestimating fecundity (i.e. reproductive rate) as well as overestimating inter-birth interval and survival of young^16,19,20,27^. Secondly, some of these approaches (e.g. inter-birth interval) cannot provide robust information on inter-annual variation or temporal trends as they require a long time series of data to estimate a single value.

Here we use a 16-year time series from bottlenose dolphins (T*ursiops truncatus*) to investigate changes in fecundity and calf survival since the population’s core habitat was designated a protected area. To account for potential variation in detection and the issues of misclassification, we use an open robust design multistate model with state uncertainty^26,28^. However, as females in this population can give birth throughout the study period we apply this model with an extension that accounts for seasonal effects. This model allows both arrivals and departures during secondary occasions and change in female state. Previous studies suggest that this population is increasing^29^, with increasing juvenile but stable adult survival^22,30^. Therefore, we aimed to determine whether there was evidence that changes in fecundity or calf survival were contributing to this increase in population abundance.

## Results

Fifty-nine reproductively mature females were sighted during the May to September 2001 to 2016 study period (annual mean = 23, SD = 10), on between one and 17 trips each year (annual mean = 5, SD = 3). These females were known to produce a total of 112 calves (annual mean = 7, SD = 3). Of these, 64 calves were seen in their year of birth (annual mean = 4, SD = 4), 87 calves were seen as 1 year olds (annual mean = 5, SD = 3) and 49 as 2 year olds (annual mean = 3, SD = 3). A minimum of zero and maximum of five calves (mean = 2, SD = 1) were born to each reproductive female. Thirteen females were seen every year since birth and had their first known calf from 6 to 14 years old (median = 9). In eight cases, a calf was assumed to have died in its first year, based upon repeated sightings of females in the following year without a calf. In all eight cases, the female was seen with a new calf two years later. A two-year inter-birth interval was also recorded for two females whose previous calves (n = 3) survived.

Analysis of these sightings histories revealed two top models that were within 0.3 AICc scores and accounted for 50% of the AICc weight (Table 1). Both specified a state effect on survival and included variation in transition probabilities and the proportion of females occupying different states over time and between states. The only difference between the two top models was that the first model (lowest AICc) included a state effect on the probability that a female was released in a specific state (*π*). However, the *β*-estimates suggested this was not estimable (*π*^*c*^ SE = 0.00), therefore we chose to present the results from the simpler second model, which had fewer parameters (Table 1).

**Table 1:** Model selection results for the open robust design multistate models with state uncertainty and seasonal effects estimating reproductive rates and calf survival of bottlenose dolphins in the SAC between 2001 and 2016.

Model^a^	K	ΔAICc	AICc weight
*S*(*s*,.), *Ψ* (*s*,*T*), *π*(s,.), *ω*(*s*,*T*), *p*(.,*t*^2^), *δ*(*s*,.), *e*(.,*t*), d(.,t), *α*(A,t^2^), *c*(.,.)	26	0.00	0.265
***S*(*s***,.**)**, *Ψ* **(*s***,***T*)**, ***π*(**.,.**)**, ***ω*(*s***,***T*)**, ***p*(**.,***t***^**2**^**)**, ***δ*(*s***,.**)**, ***e*(**.,***t*)**, **d(**.,**t)**, ***α*(A**,**t**^**2**^**)**, ***c*(**.,.**)**	**25**	**0**.**23**	**0**.**236**
*S*(.,.), *Ψ* (*s*,*T*), *π*(.,.), *ω*(*s*,*T*), *p*(.,*t*^2^), *δ*(*s*,.), *e*(.,*t*), d(.,t), *α*(A,t^2^), *c*(.,.)	23	1.21	0.145
*S*(*s*,*T*), *Ψ* (*s*,*T*), *π*(.,.), *ω*(*s*,*T*), *p*(.,*t*^2^), *δ*(*s*,.), *e*(.,*t*), d(.,t), *α*(A,t^2^), *c*(.,.)	26	1.32	0.137
*S*(*s*,.), *Ψ* (*s*,*T*), *π*(.,.), *ω*(*s*,*T*), *p*(.,*t*^2^), *δ*(*s*,*t*^2^), *e*(.,*t*), d(.,t), *α*(A,t^2^), *c*(.,.)	27	1.63	0.118
*S*(*s*,.), *Ψ* (*s*,*T*), *π*(.,.), ωs*T), *p*(.,*t*^2^), *δ*(*s*,.), *e*(.,*t*), d(.,t), *α*(A,t^2^), *c*(.,.)	24	2.19	0.089
*S*(*s*,.), *Ψ* (*s*,.), *π*(.,.), *ω*(*s*,*T*), *p*(.,*t*^2^), *δ*(*s*,.), *e*(.,*t*), d(.,t), *α*(A,t^2^), *c*(.,.)	24	5.25	0.019
*S*(*s*,.), *Ψ*(s*T), *π*. (.,.), *ω*(*s*,*T*), *p*(.,*t*^2^), *δ*(*s*,.), *e*(.,*t*), d(.,t), *α*(A,t^2^), *c*(.,.)	27	6.50	0.010
*S*(*s*,.), *Ψ* (*s*,*T*), *π*(.,.), *ω*(*s*,.), *p*(.,*t*^*2*^), *δ*(*s*,.), *e*(.,*t*), d(.,t), *α*(A,t^*2*^), *c*(.,.)	24	6.76	0.009

The best fitting model is in bold.

^a^Where *S* = female survival probability, *Ψ* = transition probability or breeding probability, *π* = probability that a female was released in a specific state, *ω* = proportion of females in a specific state, *p* = recapture probability, *δ* = probability of correctly classifying the state of a female, *e* = probability of entry to the study area, d = probability of departure from the study area, *α* = the probability that the attribute to assign the state (i.e. calf) has appeared (i.e. calving probability) and *c* = the probability that the attribute allowing the state to be determined (i.e. calf) still exists (i.e. weaning probability).

**Figure 1:**
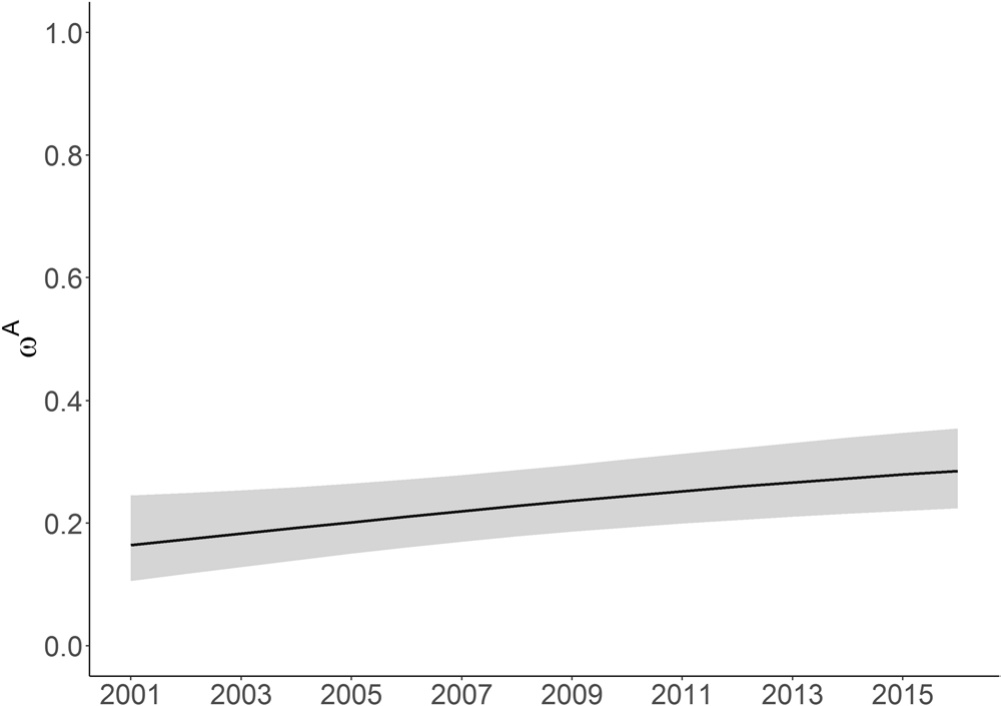
Proportion of females with newborn calves (*ω*^*A*^, the unconditional reproductive rate) from 2001 to 2016 (with 95% confidence intervals).

**Figure 2:**
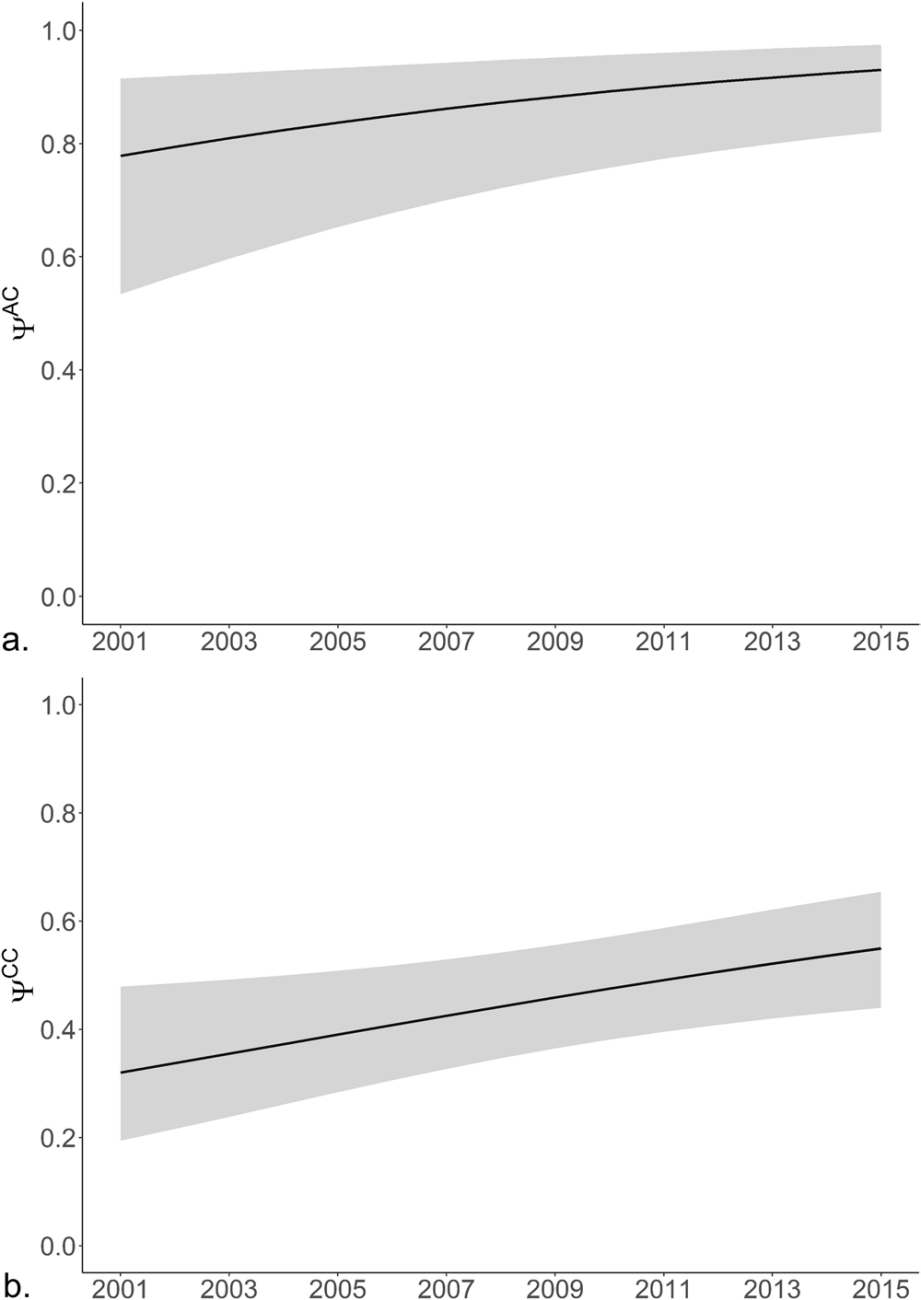
Transition probability from (**a**) a female with a newborn calf in one year to a one year old calf in the subsequent year (*Ψ*^*AC*^, first year survival) and (**b**) a female with a one year old calf in one year to female with a two year old calf in the subsequent year (*Ψ*^*CC*^, second year survival) (with 95% confidence intervals).

**Figure 3:**
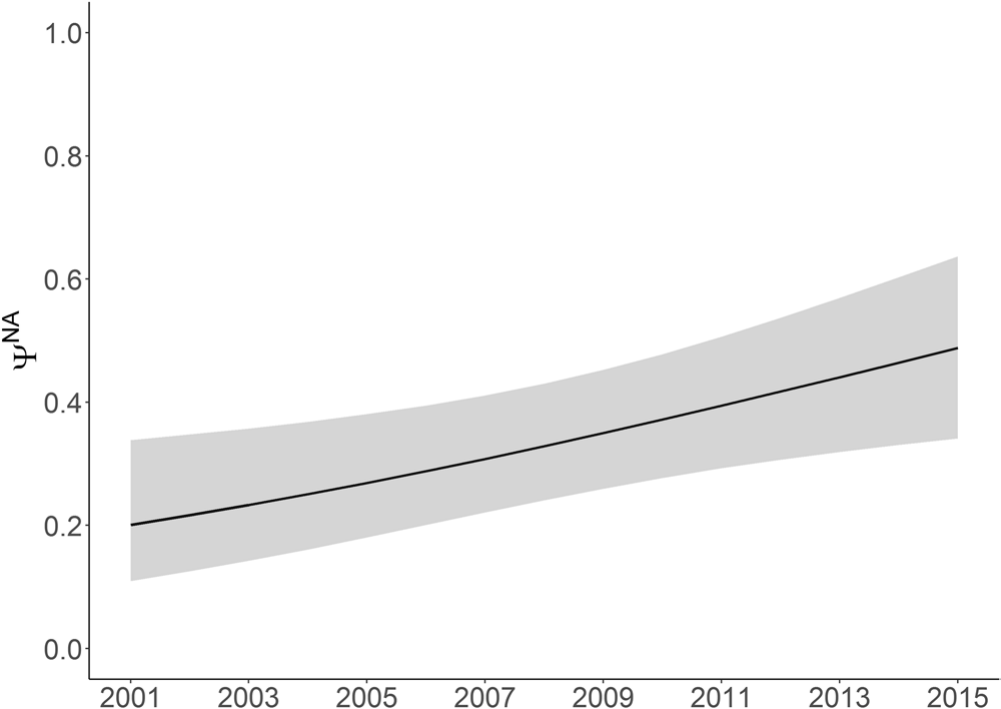
Transition probability from a non-breeder to a female with a newborn calf (conditional reproductive rate, *Ψ*^*NA*^) (with 95% confidence intervals).

This model highlighted a significant increase (*β* = 0.11, 95% CI = 0.04–0.18) in the proportion of females with newborn calves (unconditional reproductive rate, *ω*^*A*^) from 0.16 (95% CI = 0.11–0.24) in 2001 to 0.28 (95% CI = 0.22–0.36) in 2016 (Fig. 1). There was also a significant increasing trend (*β* = 0.09, 95% CI = 0.02–0.17) in each of the transition probabilities. The probability of transitioning from a female with a newborn calf in year *t* to an older calf in year *t* + 1 (first year survival, *Ψ*^*AC*^) increased over the study period from 0.78 (95% CI = 0.53–0.92) in 2001 to 0.93 (95% CI = 0.82–0.98) in 2016 (Fig. 2a). Although second year survival (transition probability from a female with an one year old calf in year *t* to a two year old calf in year *t* + 1, *Ψ*^*CC*^) remained lower, it also showed an increase from 0.32 (95% CI = 0.19–0.48) to 0.55 (95%CI = 0.44–0.65) (Fig. 2b). In addition, the transition probability from non-breeder to female with a newborn calf (conditional reproductive rate, *Ψ*^*NA*^) also increased (Fig. 3). Models without annual trends in either reproductive rate or transition probability had less support (Table 1) providing further evidence of increasing trends in these demographic rates.

There was no significant difference in apparent annual survival of females that were non-breeders (*S*^*N*^ = 0.96, 95% CI = 0.89–0.99) or females with newborn (*S*^*A*^ = 0.88, 95% CI = 0.77–0.94) or older calves (*S*^*C*^ = 0.98, 95% CI = 0.90–0.996) (see Supplementary Information Table S1 for *β*-estimates). However, the model with the lowest AIC suggested that survival of females with older calves was significantly higher than survival of females with newborn calves (*β* = 2.09, 95% CI = 0.13–4.05), but after relevelling again showed no significant difference between survival of non-breeders and females with older (*β* = −0.83, 95% CI = −1.34–3.00) or newborn calves (*β* = −1.26, 95% CI = −2.72–0.19) (Supplementary Information Table S2 and Fig. S3). This was the only difference in the results of the two top models.

**Figure 4:**
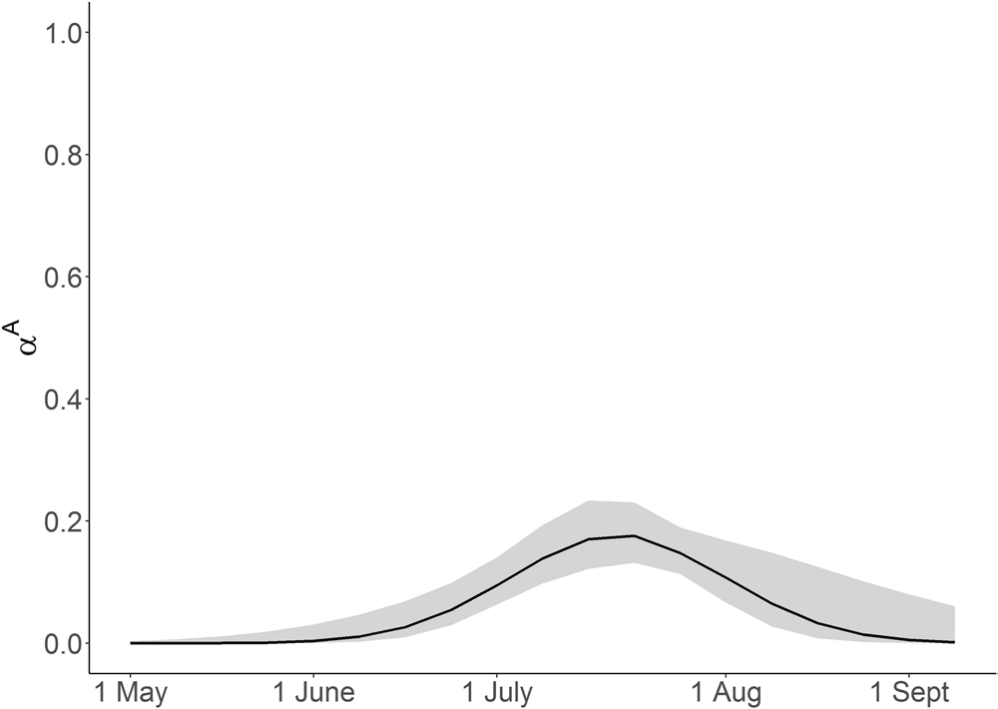
The probability that the attribute (i.e. calf) used to assign the state has arrived (*α*), i.e. the calf has been born. Showing secondary capture occasions (i.e. weeks) from May to September (with 95% confidence intervals).

As expected the chosen model suggested a peak in calving during July and August (Fig. 4). Additional model results and all associated *β*-estimates can be found in supplementary information.

## Discussion

Fecundity and early survival have impacts on population dynamics, but can be difficult to accurately estimate in highly mobile species where every birth cannot be recorded, and where births are asynchronous. We used a novel mark-recapture model that accounts for misclassification of female reproductive state and allows change of female state within the study period to estimate annual reproductive rates and first and second year calf survival. This provides the first estimates of trends in reproductive rate and calf survival for a cetacean population using a protected area, while identifying the probable vital rates affecting recent increases in abundance.

We found that the unconditional reproductive rate in this bottlenose dolphin population has significantly increased over the 16 years of this study. Comparisons with reproductive rates in other bottlenose dolphin populations are difficult as methods vary and do not account for imperfect detection. This may be why, although estimates from early in the time period fall within those reported in other populations (0.14 to 0.27^13,15,16^), our current estimate is higher than reported elsewhere. As expected, the average reproductive rate in this study (0.23, SE = 0.01) is higher than if estimated using the most common method, calculating the proportion of reproductive females with newborn calves (this study: mean = 0.14, SE = 0.03; or from another part of this population’s range on the south coast of the Moray Firth: mean = 0.16, SE = 0.04^31^). However, it is similar to an estimate modelled from the inter-birth interval for the entire east coast of Scotland population (0.22, 95% CI 0.22–0.25)^18^. Whilst the approach used by Arso Civil, *et al*.^18^ was able to take account of individual and temporal heterogeneity in re-sightings, neither this or the most commonly used method can be used to explore temporal variation in reproductive rates. The method we use here overcomes this constraint, and should provide an important tool assessing trends in reproductive rates. We encourage others to try this method, allowing direct comparisons between populations. This technique also accounts for the seasonal nature of births which is likely to be common in other populations, and the modelled predictions of a peak in births in July and August was similar to findings in a previous study using a different method^32^.

Accurately estimating early calf survival from photographic sightings of calves can be difficult because calves may have low sighting probability or die before being observed. Furthermore, young individuals tend to have fewer marks for identification, and may be difficult to follow given that they can become more independent at different ages. Here, we used transition probabilities for females in different reproductive states as a proxy for early calf survival. These results suggest that both first and second year survival have increased over the study period, although survival of older calves remains lower. Other studies of trends in calf survival in cetaceans are rare. Currey, *et al*.^33^ found a decline in first year calf survival over two time periods in another temperate bottlenose dolphin population, which they suggest was a result of cold freshwater inflow from a hydroelectric station and a key factor in that population’s decline. Comparing results with a previous study across the entire range of this population^22^ suggests that calf survival may be different within the protected area, as our study shows increasing early calf survival, and higher first year but lower second year calf survival within the SAC. This could indicate that the SAC is a source for the bottlenose dolphin population on the east coast of Scotland, including early dispersal of second year calves. However, this disparity may also be due to methodological differences. Accounting for imperfect detections in this current study could explain the increasing trend and higher current survival estimates for first year calves. Indeed the first year calf survival estimate in the previous study (0.865, 95% CI 0.785–0.919)^22^ is similar to the average estimate over this study (0.87, SE = 0.01). In addition Arso Civil, *et al*.^22^ only included calves where the mothers were seen in the year the calf was born and the three subsequent years. This may have selected for well-known experienced mothers, overestimating second year survival (0.981, 95% CI 0.797–0.998)^22^, that was also higher that their estimate of adult survival (0.948 95% CI 0.933–0.959)^22^. In future, using data from across the entire range of this population in the current model could disentangle these issues, but this is currently constrained by more limited long-term data collection outside the SAC.

Although, first year survival estimates in the early phase of our study fell within the reported range for other *Tursiops* spp. populations (0.70 to 0.86^15,16,20,34,35^), estimates from more recent years were higher. Again comparisons are difficult but this may be, at least in part, due to the lack of shark predation which can be a key mortality risk for young calves in other areas^36–38^. Our latest second year survival estimate (0.55, 95% CI 0.44–0.65) was lower than previous estimates of second year survival from Australian waters (0.82)^20^ but similar to the survival rate for older, weaned calves in other areas (0.54^39,40^). Our lower estimate could result from a decreased probability of detecting older calves that spend less time with their mothers^41^, but our model choice should have accounted for this. There is evidence of calf mortality due to infanticide in this population, but all the calves known to be killed by conspecifics were in their first year of life^42^. Steiner and Bossley^39^ suggested this higher mortality of weaned calves in Australia was a result of anthropogenic activities (e.g. boat strikes and entanglement), however, there has been no evidence of similar fatal events in our study area. Lactation is thought to last for 18 months to 2 years and solids have been found in the stomachs of calves from ~6 months old^43^. Therefore, reduction in provisioning by the female as calves get older could result in a decrease in condition and increase in mortality compared to newborn calves.

Understanding the demographic causes of changes in population status can provide valuable information for conservation and management^10^. Recent empirical evidence suggests that our study population has been increasing^29,44^, adult survival has remained stable^22,30^ and juvenile survival has increased^22^. This study provides additional empirical data that suggest that the growth of this population is the consequence of increased fecundity and first and second year calf survival. Previous modelling exercises also suggested that high reproductive rates and increased juvenile survival drove recent increases in Steller sea lions (*Eumetopias jubatus*) in Alaska^45^, and that reproduction was the key vital rate affecting contrasting population trajectories in two tropical bottlenose dolphin populations^46^. Evaluation of the contribution of the recent conservation management to these positive trends now requires a better understanding of the key intrinsic and/or extrinsic drivers that have influenced reproduction and calf survival in this population. Fecundity can vary with age^3^ and increasing reproduction may be the result of an increase in the number of prime breeding age females. However, it can also vary with food availability^20,47,48^. Data on changes in female age structure are not currently available for our study population, although ongoing photogrammetry work^49^ may shed light on this question in future. Similarly, understanding of trends in food availability is constrained by limited information on diet in this population and on the availability of suitably fine-scale data on variation in abundance of potential prey. Work is underway to investigate abundance and distribution of likely prey in key areas used for foraging by dolphins.

Investigating first and second year calf body condition could help identify the factors driving the increase in early survival. Photogrammetric evidence from this study population suggests that calves that survived their first winter were significantly longer than those that died^49^. This has also been shown in an Indian Ocean population where poor calf condition was the main cause of mortality^50^. In New Zealand waters, bottlenose dolphin calf mortality was affected by timing of calving^19,47^ and female heterogeneity, with females showing consistent differences in survival of their calves^19^. These could be proxies for calf condition, as calf condition is likely linked to maternal experience and condition^50^.

Our results also provided some evidence for a cost of reproduction in bottlenose dolphin females, as females with newborn calves had a slightly higher mortality than those with older calves. Whether this was significant depended on the choice between two top models, which were very similar. This uncertainty may largely be due to the limited amount of data available. However, it could be the result of heterogeneity in individual female quality, which can also influence variability in the cost of reproduction, with no reproductive costs for some individuals or higher survival for breeders in other cases^8^. In addition, reproductive costs are affected by both extrinsic (e.g. habitat quality, prey availability) and intrinsic factors (e.g. female age, calf sex, timing of birth)^7^, which can interact^51^ and complicate interpretation. Further work is required to investigate these hypotheses.

Marine protected areas (MPAs) have been established worldwide to protect marine mammals^52^, although there is limited evidence on their effectiveness as management and conservation tools for this group^53^. This study presents a rare example of empirical evidence of a positive trend in reproduction and survival in a cetacean population using an MPA (see ref.^53^ for another example). Whether this is a consequence or coincidental to the establishment of the protected area status is unknown. However, in combination with previous work showing the population is increasing^29,44^, these results suggests this small bottlenose dolphin population can be considered a conservation success story.

## Methods

### Study Site and Data Collection

The study used data on bottlenose dolphins using the Moray Firth Special Area of Conservation (SAC) (92/43/EEC) on the east coast of Scotland. The SAC was designated in 2005 and requires any activity that could have a potential significant effect on the SAC’s conservation objectives (i.e. to avoid deterioration of the bottlenose dolphin habitat, or significant disturbance to the population) to undertake an appropriate assessment that demonstrates that the activity does not impact these objectives^54,55^. This bottlenose dolphin population has been studied intensively since 1989 as part of a long-term individual-based demographic study, principally using photo-identification^29,56,57^. The population size was estimated at 195 (95% highest posterior density interval 162–253) in 2006^58^ and is increasing^29,44^.

Sightings of females and calves (newborn to 2 years old) were available from between 19 and 39 annual photo-identification surveys carried out from May to September 2001 to 2016 (total of 416 surveys). This period was chosen because a change in survey protocol in 2001 led to an increase in re-sightings rates^29^. This meant that the majority of calves could be associated with known females (e.g. seen with the calf on at least two occasions and/or in echelon position, consistently surfacing alongside the females’ dorsal fin with no other adjacent females). The year of birth was estimated using the presence and clarity of foetal folds (vertical creases down their sides from their position in the womb, which fade over time), skin colour and relative size^41^. Sightings of females were included once they had been observed with a calf, either during or prior to this study, and were therefore known to be reproductively mature. Only the best quality pictures were used in the analyses and identifications were confirmed by two experienced researchers^56,58^.

### Estimating fecundity and calf survival

Fecundity and calf survival were estimated using an open robust design multistate model with state uncertainty and seasonal effects^59,60^. The open robust design multistate model with state uncertainty accounts for misclassification or uncertainty in breeding status (e.g. the female is classified as a non-breeder when she is a breeder, because she is observed but her calf is not)^26^. This model applies the robust design approach^61^ to use information from multiple sightings of females (with or without a calf) within a year to estimate the probability that a calf is detected if it is present. This detection probability is then used to adjust breeding probabilities (for full details of the state uncertainty model see ref.^26^). In addition, this new model with seasonal effects also incorporates two new parameters, which allow a change in female state (i.e. arrival or departure of a calf) during secondary occasions^59,60^. The first is alpha (*α*), the probability that the attribute (in this case the calf) used to assign the state has arrived. This provides a measure of the probability that the calf has been born^59,60^. The second is *c*, the probability that the attribute allowing the state to be determined still exists, i.e. the probability that the calf is weaned^59,60^.

In this study females could be assigned to one of three states: N = non-breeders, A = females with newborn calves and C = females with older (1 or 2 year old) calves. However, N was never observed with certainty as a calf may not be detected, and females without a calf cannot unambiguously be classified as non-breeders^26^. Therefore, the model also included an uncertain state or unobservable event (u), which was assigned to females when they were not observed with a calf (i.e. a mixture of non-breeders and breeders where the calf was hidden or obscured, not photographed, missing or already dead). Each survey year from May to September (i.e. primary occasion) was divided into weeks (i.e. secondary capture occasions). Sightings of each female were pooled into these weekly secondary capture occasions within each primary occasion.

The model parameters of interest were estimates of the proportion of females within the study area that have a newborn calf (*ω*^*A*^, the unconditional reproductive rate) and the transition probability from a non-breeder in year *T* to female with a newborn calf in year *T* + 1 (*Ψ*^*NA*^, conditional reproductive rate). The transition probability of a female with a newborn calf in year *T* to an older calf in year *T + *1 (*Ψ*^*AC*^) equates to first year calf survival (newborn to 1 year old). Finally, the probability that a female with an older calf (1 year old) in year *T* still had an older calf (2 year old) in year *T* + 1 (*Ψ*^*CC*^) provides a proxy for second year calf survival (1 to 2 years old). The model also provides the apparent annual survival of females (S) in each state and their calving probability (*α*) (for full details of other available model parameters see refs^26,28,59,60,62^).

Following Taylor, *et al*.^63^, we a priori identified a limited number of models consistent with bottlenose dolphin biology while being careful not to over-parameterize models (for example we did not explore annual or weekly variation, but instead fit a linear trend). We fitted parameters with a state effect (s), linear or quadratic time trend across primary occasions (*T* or *T*^2^), linear or quadratic time trend across secondary occasions (*t* or *t*^2^) or no variation between states and/or across time (.). For female survival (S), models with and without a state effect were included to explore potential costs of reproduction. In addition, this parameter was modelled without annual variability, as adult survival was previously found to be stable within this population^22,30^. However, to confirm this, a linear trend was added to the best fitting model. We were specifically interested in temporal trends and therefore explored variation in transition probabilities (*Ψ*) between states and the proportion of the population in each state in each year (*ω*). Models were therefore constructed with a state effect and a linear time trend across primary occasions for these parameters. To determine if linear trends varied for different states, an interaction between state and time trend (s*T) was included in some models. In addition, to determine if there was a linear time trend in transition probabilities and reproductive rates, the best model was run with no variation across time for both *Ψ* and *ω*. We expected no temporal variability in the probability that a female was released in a specific state (*π*), so considered models with no time variation but with and without a state effect. All models contained no state effect for capture probabilities (*p*). We expected this parameter to be independent of female state as our survey area is not a specific nursery ground (i.e. newborn calves have been observed throughout the range of this population^32^) and our field protocols aim to photograph all the individuals in a group. However, we did include a quadratic time trend across secondary occasions to capture a seasonal pattern in recapture probabilities over the summer. For the probability of correctly classifying the state of a female (*δ*), we considered models with a state effect and with no time trend. Female state may be misclassified but there is unlikely to be a trend throughout secondary occasions. However, a quadratic time trend across secondary occasions was added to the best fitting model for confirmation. Again, as the survey area is not a specific nursery area, we suspect there would be no state effect for the probability of entry to the study area (pent: *e*) or probability of departure from study area (d), and models were therefore built without a state effect. As there is an apparent summer increase in dolphins in our study area^57^, linear time trends across secondary occasions were included for these parameters. In this bottlenose dolphin population newborn calves were observed from June to October, with a peak in late summer^32^ (Supplementary Information Fig. S1). Therefore, all the models included a quadratic time trend for *α*^*A*^ across secondary occasions. Finally, all the models had a constant *c* as additional states for older weaned calves (i.e. calf is no longer seen in infant/echelon position with a marked decrease in mother-calf association^20^) were not included to avoid over-parameterising the model. Additionally, there is a lack of synchrony in time of weaning in dolphins, as bottlenose dolphin calves can be weaned at 2 to 9 years old^19,20,41^, which limits the use of this parameter.

For the misclassification probability, we presumed that all calves were correctly assigned to their mothers, and therefore a non-breeder could not be wrongly identified as a breeder (*δ*^*N*^ = 0). We also assumed that a female could not give birth in two consecutive years (*Ψ*^*AA*^ = 0), as that has never been reported in temperate bottlenose dolphins^19,32^. Finally, a female could not transition from a non-breeder directly to having an older calf (i.e. in consecutive years) (*Ψ*^*NC*^ = 0).

Analyses were carried out in R^64^ within the package RMark^65^ to construct models in MARK^66^. Model selection was conducted using Akaike’s Information Criterion (AIC)^67^, adjusted for small sample size (AICc), and AICc weights^68^. Beta-estimates (*β*) and 95% confidence intervals (CI) were used to assess effect sizes (i.e. significant when the 95% CI do not overlap with zero).

## Supplementary information

Supplementary Information

Full Dataset
