# Supplementary material for: Increasing trends in fecundity and calf survival of bottlenose dolphins in a marine protected area
Source: Sci Rep. 2019 Feb 11;9:1767. doi: 10.1038/s41598-018-38278-9 (PMC6370779; doi:10.1038/s41598-018-38278-9)
Supplement: Supplementary file 1 — Supplementary Information [file 41598_2018_38278_MOESM1_ESM.pdf]

Increasing trends in fecundity and calf survival of bottlenose dolphins in a marine protected area

Barbara J. Cheney, Paul M. Thompson and Line S. Cordes

Supplementary Information

Table S1. Beta estimates to assess effect sizes from the best fitting open robust design multistate model with state uncertainty and seasonality ($S(s,.)$, $\psi(s,T)$, $\pi(.,.)$, $\omega(s,T)$, $p(.,t^2)$, $\delta(s,.)$, $e(.,t)$, d(.,t), α(A,t^2), $c(.,.)$).

Parameter	Estimate	Standard Error	Lower Confidence Interval	Upper Confidence Interval
S:(Intercept)	1.9787485	0.3968469	1.2009286	2.7565684
S:stratumC	1.8702147	0.9732294	-0.0373149	3.7777443
S:stratumN	1.2093010	0.7539234	-0.2683888	2.6869909
Psi:Time	0.0952297	0.0365711	0.0235503	0.1669090
Psi:stratumC:tostratumA	-1.6118133	0.4462427	-2.4864490	-0.7371775
Psi:stratumN:tostratumA	-1.3810308	0.3633328	-2.0931631	-0.6688985
Psi:stratumA:tostratumC	1.2567008	0.5714552	0.1366486	2.3767530
Psi:stratumC:tostratumC	-0.5706642	0.3959615	-1.3467486	0.2054203
pi:(Intercept)	38.3383480	0.0000000	38.3383480	38.3383480
Omega:(Intercept)	-1.1736174	0.3689554	-1.8967700	-0.4504647
Omega:stratumC	0.6203760	0.1551754	0.3162323	0.9245197
Omega:Time	0.1072542	0.0368062	0.0351140	0.1793944
p:(Intercept)	0.1080194	0.1314513	-0.1496252	0.3656640
p:Time	-0.1782940	0.0320695	-0.2411503	-0.1154378
p:I(Time^2)	0.0091871	0.0018190	0.0056219	0.0127523
Delta:(Intercept)	0.9135894	0.2317021	0.4594533	1.3677254
Delta:stratum	1.6277615	0.2841064	1.0709130	2.1846099
pent:(Intercept)	-0.7960786	0.1342179	-1.0591456	-0.5330116
pent:Time	-0.2187439	0.0221562	-0.2621700	-0.1753177
d:(Intercept)	-6.9470141	0.7037412	-8.3263468	-5.5676814
d:Time	0.3266308	0.0573833	0.2141596	0.4391020
alpha:(Intercept)	15.3197240	485.0930400	-935.4626600	966.1021000
alpha:A:Time	1.8638345	0.6274251	0.6340812	3.0935877
alpha:A:I(Time^2)	-0.0872796	0.0311053	-0.1482459	-0.0263132
c:(Intercept)	-7.2512932	0.9995006	-9.2103145	-5.2922719
Releveled Data				
S:(Intercept)	3.1880545	0.5477579	2.1144489	4.2616601
S:stratumA	-1.2093091	0.7539219	-2.6869959	0.2683778
S:stratumC	0.6609036	1.0441931	-1.3857150	2.7075222

Table S2. Beta estimates to assess effect sizes from the lowest AICc open robust design multistate model with state uncertainty and seasonality ($S(s,.)$, $\psi(s,T)$, $\pi(s,.)$, $\omega(s,T)$, $p(.,t^2)$, $\delta(s,.)$, $e(.,t)$, d(.,t), $\alpha(A,t^2)$, $c(.,.)$).

**Parameter**	**Estimate**	**Standard Error**	**Lower Confidence Interval**	**Upper Confidence Interval**
S:(Intercept)	1.9280338	0.3756862	1.1916888	2.6643788
S:stratumC	2.0909423	1.0016086	0.1277893	4.0540952
S:stratumN	1.2630301	0.7435160	-0.1942613	2.7203216
Psi:Time	0.0961188	0.0366565	0.0242721	0.1679656
Psi:stratumC:tostratumA	-1.6244524	0.4481690	-2.5028636	-0.7460412
Psi:stratumN:tostratumA	-1.3820527	0.3641337	-2.0957548	-0.6683506
Psi:stratumA:tostratumC	1.2597948	0.5699132	0.1427649	2.3768248
Psi:stratumC:tostratumC	-0.5915601	0.3973685	-1.3704024	0.1872822
pi:(Intercept)	38.5157680	0.0000000	38.5157680	38.5157680
pi:stratumC	-43.3396360	0.0000000	-43.3396360	-43.3396360
Omega:(Intercept)	-1.1734839	0.3689755	-1.8966758	-0.4502920
Omega:stratumC	0.6202913	0.1551815	0.3161356	0.9244470
Omega:Time	0.1072618	0.0368103	0.0351137	0.1794099
p:(Intercept)	0.1090890	0.1314775	-0.1486068	0.3667849
p:Time	-0.1787629	0.0320768	-0.2416335	-0.1158922
p:I(Time^2)	0.0092212	0.0018194	0.0056551	0.0127873
Delta:(Intercept)	0.9114563	0.2316483	0.4574255	1.3654870
Delta:stratumC	1.6303577	0.2840285	1.0736617	2.1870537
pent:(Intercept)	-0.7928528	0.1342744	-1.0560307	-0.5296749
pent:Time	-0.2186889	0.0221538	-0.2621104	-0.1752673
d:(Intercept)	-6.9502934	0.7033549	-8.3288691	-5.5717177
d:Time	0.3270526	0.0573221	0.2147013	0.4394039
alpha:(Intercept)	15.44563605	34.3013200	-1031.7850000	1062.6762000
alpha:A:Time	1.8645089	0.6276757	0.6342645	3.0947532
alpha:A:I(Time^2)	-0.0873223	0.0311202	-0.1483180	-0.0263267
c:(Intercept)	-7.2517309	0.9995946	-9.2109363	-5.2925255
Releveled Data				
S:(Intercept)	3.1912414	0.5532637	2.1068444	4.2756384
S:stratumA	-1.2631322	0.7436347	-2.7206562	0.1943919
S:stratumC	0.8278735	1.1084999	-1.3447863	3.0005332

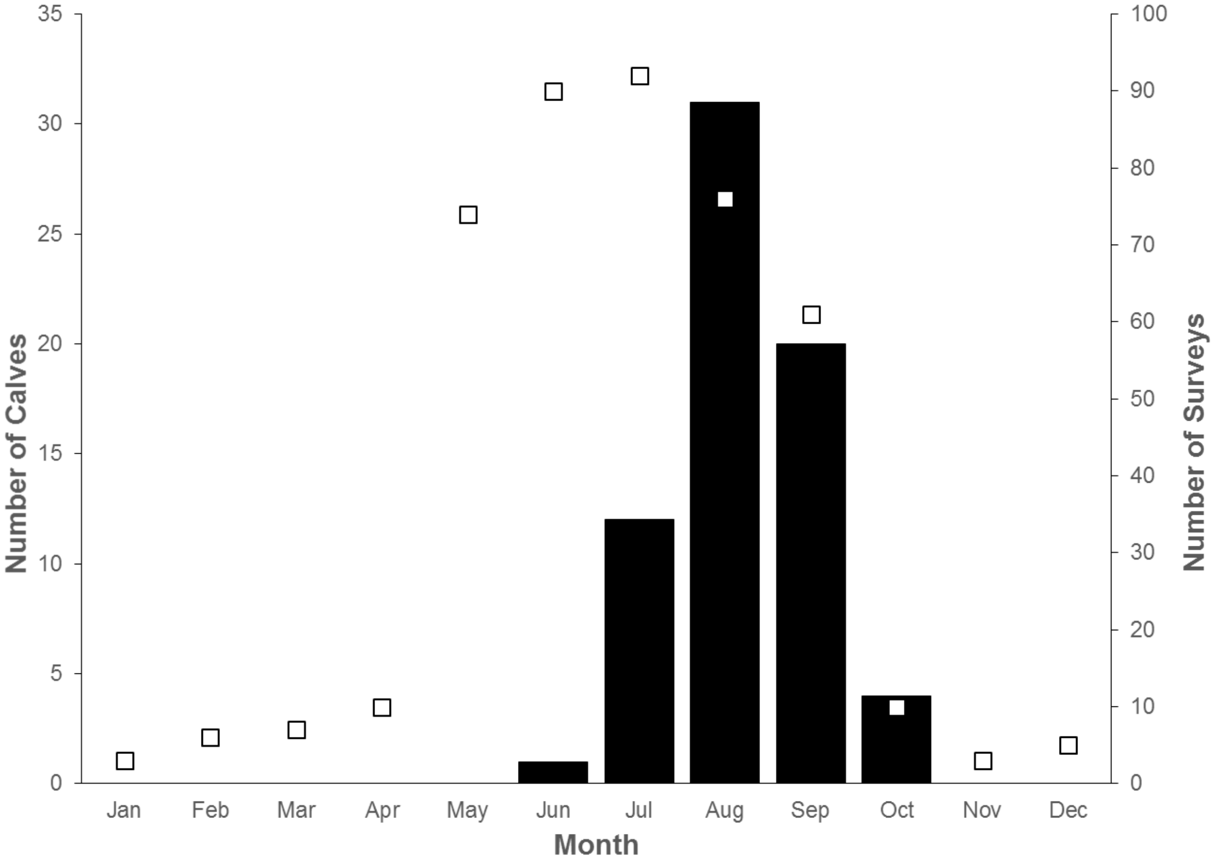

Figure S1. Seasonal changes in the timing of first sightings of females with neonates in the SAC from 2001 to 2015 (black bars), and the number of surveys carried out each month (white squares)

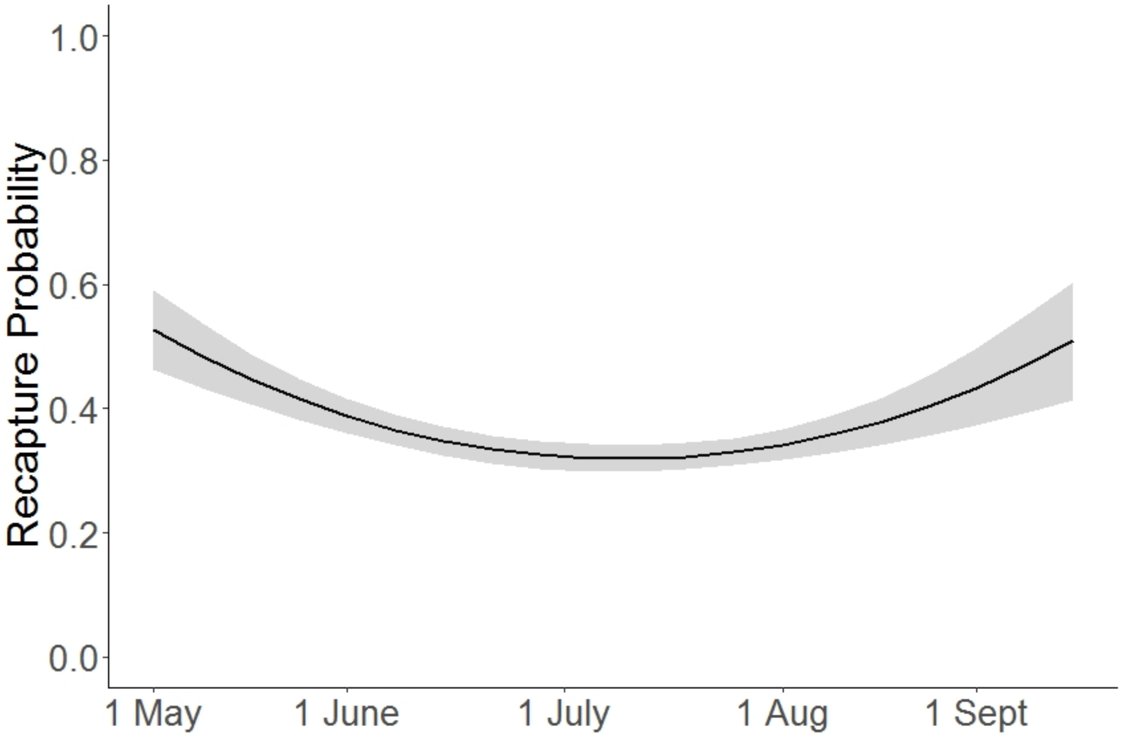

a.

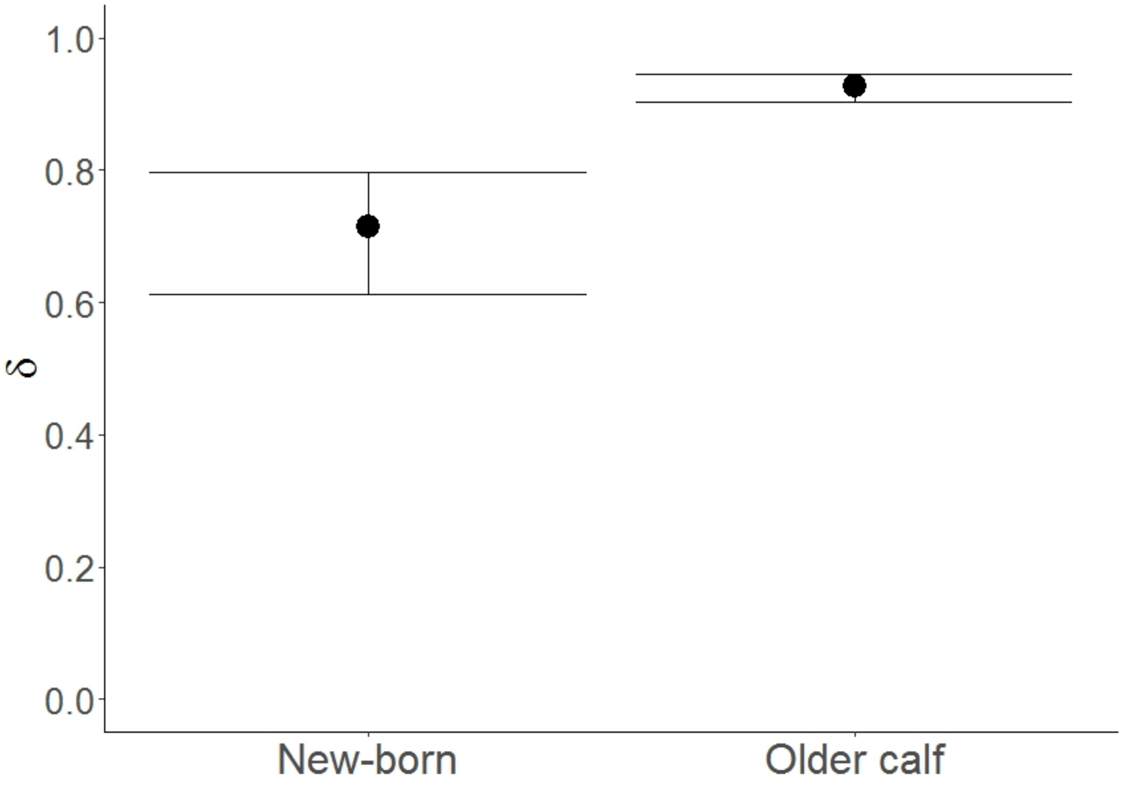

b.

Figure S2. Additional results from the best fitting open robust design multistate model with state uncertainty and seasonality ($S(s,.)$, $\psi(s,T)$, $\pi(.,.)$, $\omega(s,T)$, $p(.,t^2)$, $\delta(s,.)$, $e(.,t)$, d(.,t), α(A,t^2), $c(.,.)$ including a. recapture probability for females with new-born calves (p^A) and b. the probability that a calf was observed given that a female was with a new-born (δ^A) or older calf (1 or 2 years old, δ^C) during the summer (all with 95% confidence interval).

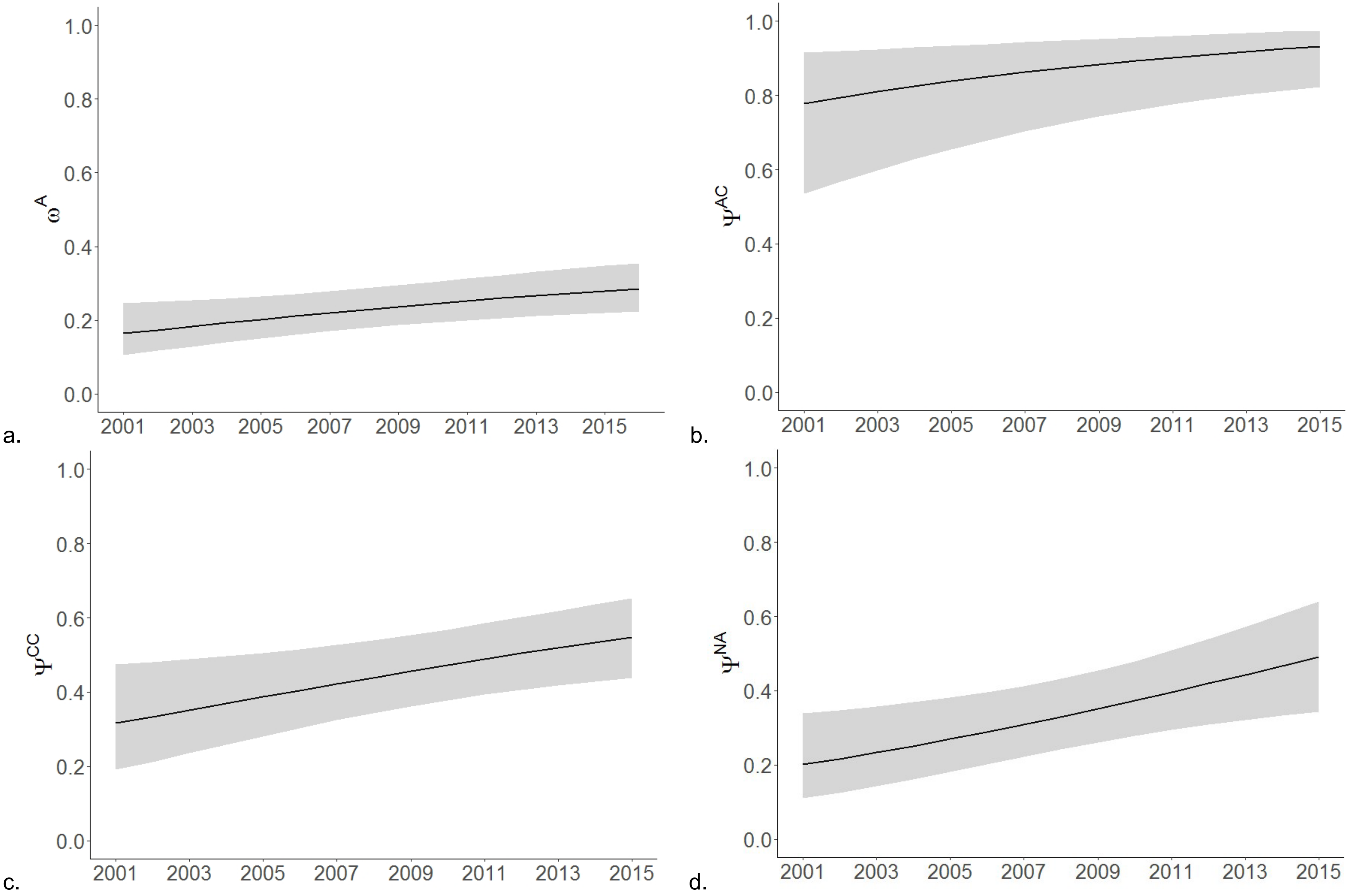

Figure S3. Results from the lowest AICc open robust design multistate model with state uncertainty and seasonality (($S(s,.)$, $\psi(s,T)$, $\pi(s,.)$, $\omega(s,T)$, $p(.,t^2)$, $\delta(s,.)$, $e(.,t)$, d(.,t), $\alpha(\mathrm{A},\mathrm{t}^2)$, $c(.,.)$) including a. proportion of females with new-born calves (ω^A, the unconditional reproductive rate), b. transition probability from a female with a new-born calf in one year to a one year old calf in the subsequent year (ψ^{AC}, second year survival), c. transition probability from a female with a 1 year old calf in one year to female with a two year old calf in the subsequent year (ψ^{CC}, second year survival), d. transition probability from a non-breeder to a female with a new-born calf (conditional reproductive rate, ψ^{NA}) (continued below)

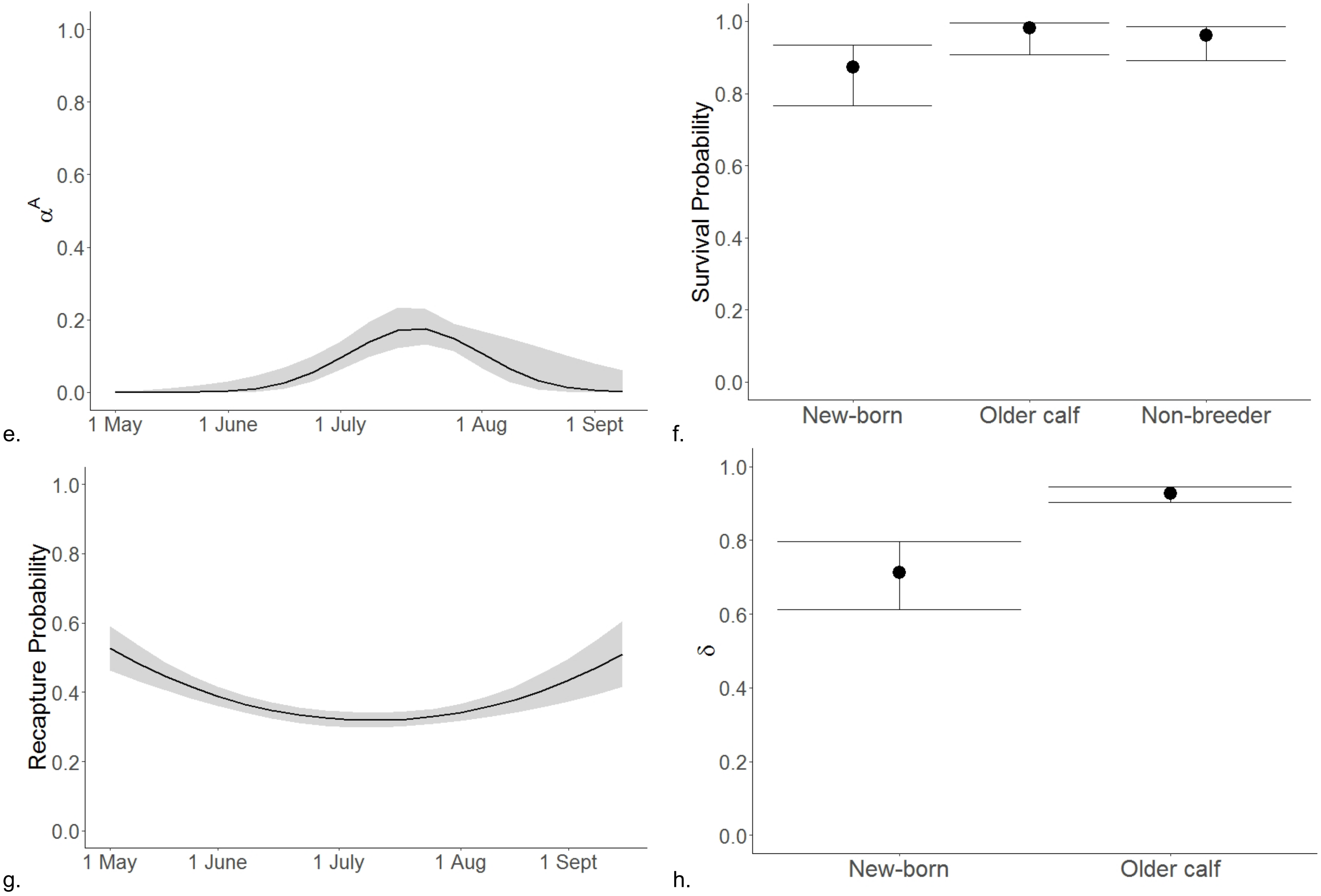

Figure S3 (cont.) e. the probability that the attribute (i.e. calf) used to assign the state has arrived (α), i.e. the calf has been born, f. survival probabilities (S) for females with new-born or older calves and non-breeding females, g. recapture probability for females with new-born calves (p^A) and h. the probability that a calf was observed given that a female was with a new-born (δ^A) or older calf (1 or 2 years old, δ^C) during the summer (all with 95% confidence interval).

R Code for our best Open Robust Design Multistate Model with State Uncertainty and Seasonal Effects.

```
install.packages("RMark")
library(RMark)

fec<-read.csv("full_data_set_2001-2016.csv", header=T, colClasses=c("character"))
head(fec)

#for time period where weeks with no sightings (weeks-1).
time.interval=c(rep(0,16),1, rep(0,16),1, rep(0,16),1, rep(0,12),1, rep(0,14),1, rep(0,19),1, rep(0,16),1, rep(0,15),1,
        rep(0,17),1, rep(0,15),1, rep(0,17),1, rep(0,16),1, rep(0,17),1, rep(0,15),1, rep(0,16),1, rep(0,18))

#process data - 'container' to store data and its attributes
#N = non-breeder to be estimated
fec.proc<-process.data(fec, model="RDMSOpenMCSeas", begin.time=2001, time.interval=time.interval, strata.labels=c("A","C","N"))

#where N = non-breeder
#A = newborn
#C = older calf (either 1 or 2)

#create design data (subtract.stratum is where you control which transition probabilities are obtained by subtraction,
#i.e. not shown, ("N", "N", "N") = A to N, C to N and N to N are obtained by subtraction,

fec.ddl=make.design.data(fec.proc, parameters=list(S=list(pim.type="time"), Psi=list(pim.type="time", subtract.stratum=c("N","N","N")),
                          pi=list(pim.type="time"), Omega=list(pim.type="time"), p=list(pim.type="time"),
                          pent=list(pim.type="time"), d=list(pim.type="time"), alpha=list(pim.type="time")))

head(fec.ddl$S)
head(fec.ddl$p)
head(fec.ddl$alpha)

#view part of design matrix (PIM)
dm=model.matrix(~time,fec.ddl$alpha)
```

```
head(dm)

#fix transition probability from newborn to newborn (A to A) to 0
fec.ddl$Psi$fix=NA
fec.ddl$Psi$fix[fec.ddl$Psi$stratum=="A" &
        fec.ddl$Psi$tostratum%in%c("A")]=0

#fix transition probability from non-breeder to older calf (N to C) to 0
fec.ddl$Psi$fix[fec.ddl$Psi$stratum=="N" &
        fec.ddl$Psi$tostratum%in%c("C")]=0

#can't estimate delta for N as not visible
fec.ddl$Delta$fix=NA
fec.ddl$Delta$fix[fec.ddl$Delta$stratum%in%c("N")]=0

##################################################################################################################
# S      = female survival probability (primary)
# Psi    = transition probability or breeding probability (primary)
# pi     = probability that a female was released in a specific state (primary)
# Omega = proportion of females in a specific state, e.g. proportion of females that breed in a certain year (primary)
# p      = recapture probability (primary and secondary)
# Delta = probability of correctly classifying the state of a female (primary and secondary)
# pent   = probability of entry to the study area. mlogit link should be used for pent  (primary and secondary)
# d      = think of the d parameter as departure (rather than remain as for phi). d = 1 - phi (from the usual model without seasonal effects). Only
time specific PIM (primary and secondary)
# alpha = the probability that the attribute to assign the state has appeared (birth of calf/pup). Must sum to 1 (primary and secondary)
# c      = the probability that the attribute allowing the state to be determined still exists (weaning probability) (primary and secondary)

#For p, Delta, pent, d, alpha, c you can look at variation over secondary occasions, so "session" is time or year in this case, and "Time" are the
within year secondary occasions
##################################################################################################################
```

```
#Best model

setwd("…………./Bestmodel")

S=list(formula=~stratum)
Psi=list(formula=~-1 + stratum:tostratum + Time)
pi=list(formula=~1)
Omega=list(formula=~stratum + Time)
p=list(formula=~Time + I(Time^2))
Delta=list(formula=~stratum)
pent=list(formula=~Time)
d=list(formula=~Time)
alpha=list(formula=~A:Time + A:I(Time^2))
c=list(formula=~1)

Bestmodel=mark(fec.proc, fec.ddl, model.parameters=list(S=S, Psi=Psi, pi=pi, Omega=Omega, p=p, Delta=Delta, pent=pent, d=d,
alpha=alpha, c=c), mlogit0=TRUE)

save(Bestmodel, file="model_output_best")

#Model selection

model.table(model.list=NULL,type="RDMSOpenMCSeas",sort=TRUE,model.name=FALSE,adjust=TRUE,ignore=TRUE)
```

Data Input for R

Our full dataset is supplied in a csv file where each row represents one female and each character represents a week (0=female not observed, u= female observed without a calf/uncertain breeding state, A = female observed with a new-born calf, C = female observed with a 1 or 2 year old calf).
